# Clinical Usefulness of Oral Supplementation with Alpha-Lipoic Acid, Curcumin Phytosome, and B-Group Vitamins in Patients with Carpal Tunnel Syndrome Undergoing Surgical Treatment

**DOI:** 10.1155/2014/891310

**Published:** 2014-01-19

**Authors:** Giorgio Pajardi, Paola Bortot, Veronica Ponti, Chiara Novelli

**Affiliations:** Hand Surgery Unit, Plastic Surgery Department, MultiMedica Holding S.p.A, Via Milanese, Sesto San Giovanni, 300-20099 Milan, Italy

## Abstract

We investigated the clinical usefulness of oral supplementation with a combination product containing alpha-lipoic acid, curcumin phytosome, and B-group vitamins in 180 patients with carpal tunnel syndrome (CTS), scheduled to undergo surgical decompression of the median nerve. Patients in Group A (*n* = 60) served as controls and did not receive any treatment either before or after surgery. Patients in Group B (*n* = 60) received oral supplementation twice a day for 3 months both before and after surgery (totaling 6 months of supplementation). Patients in Group C (*n* = 60) received oral supplementation twice a day for 3 months before surgery only. Patients in Group B showed significantly lower nocturnal symptoms scores compared with Group A subjects at both 40 days and 3 months after surgery (both *P* values <0.05). Moreover, patients in Group B had a significantly lower number of positive Phalen's tests at 3 months compared with the other study groups (*P* < 0.05). We conclude that oral supplementation with alpha-lipoic acid, curcumin phytosome, and B-group vitamins twice a day both before and after surgery is safe and effective in CTS patients scheduled to undergo surgical decompression of the median nerve.

## 1. Introduction

Peripheral nerve compression syndromes (also known as tunnel syndromes or entrapment neuropathies) are common disabling conditions that occur when nerves are compressed through bony, fibrous, osteofibrous, and fibromuscular tunnels, impairing nerve function [1]. The economic and social costs of peripheral nerve compression syndromes are high due to lost working days, change of occupation, and the need for surgical intervention [2]. From a clinical standpoint, patients with carpal tunnel syndromes can present with motor, sensory, or autonomic findings, although they most show motor and sensory complaints [1, 2].

Carpal tunnel syndrome (CTS) is the most common entrapment neuropathy in the upper extremity, with a lifetime risk of approximately 10% [3]. CTS is caused by the compression of the median nerve, which temporarily causes conduction blocks within large myelinated nerve fibers, along with the blockage of capillary blood flow to the median nerve through the swelling of the ensheathing synovial tissue of the tendons. The primary symptoms of classic CTS involve numbness and tingling with or without pain in at least two of the median nerve innervated fingers [3]. Symptoms are often aggravated during sleep and in daytime caused by static or repetitive hand function. The vast majority of cases of CTS are either idiopathic or spontaneous, presenting bilateral symptoms in over 60% of the patients [4]. Common conditions related to secondary CTS include high energy wrist traumas, endocrine disorders (e.g., diabetes mellitus and hypothyroidism), pregnancy, rheumatoid arthritis, anomalous carpal tunnel structures, and occupational factors such as repetitive motion or exposure to vibrating tools [5]. Patients with CTS may experience quite variable sensory symptoms and pain [3–5]. Paresthesia and pain may extend proximal to the wrist in nearly 40% of cases, whereas predominant involvement of the dorsum of the hand has been reported to occur in 11% of the cases [3].

The mainstay of definitive therapy for severe CTS is surgery [6], either with or without conservative approaches [7] aimed at limiting ischemia-induced changes in the nerve and reducing inflammation of the flexor tenosynovium. However, most of the conventional agents currently used in the management of CTS are designed to hit a single target in the pathophysiology of nerve compression. Unfortunately, the physiological and mechanistic deregulations responsible for CTS initiation and perpetuation implicate a number of mechanisms, so that it appears evident that multitargeted approaches are required to overcome this disabling entrapment neuropathy [6, 7].

Alpha-lipoic acid [8, 9], curcumin [10, 11], and B-group vitamins [12–14] have been shown to exert significant anti-inflammatory, antioxidant, and neuroprotective effects in the peripheral nerves. Recently, a form of curcumin coated with phospholipids (curcumin phytosome) has greatly improved its oral bioavailability (~29 times greater than that of traditional curcumin) [15].

In this report, we sought to analyze the clinical usefulness of oral supplementation with a combination product (Axin C) containing such active compounds in a sample of CTS patients who were scheduled to undergo surgical decompression of the median nerve. The primary aim of this study was to compare the magnitude of symptoms reduction (hyperalgesia, paresthesia, nocturnal symptoms, and limitations to everyday life) in CTS patients who received oral supplementation both before and after surgery compared with those who did not. Secondary endpoints were the numbers of positive and negative results in Tinel's and Phalen's tests, compliance, and patients' subjective satisfaction with oral supplementation.

## 2. Methods

### 2.1. Participants

This research was designed as a prospective, observational study of 180 consecutive CTS patients (44 males and 136 females, mean age: 57.9 ± 14.8 years) who were scheduled to undergo surgical decompression of the median nerve at the IRCCS MultiMedica (Sesto San Giovanni, Italy). The enrollment period was 6 months, with a planned minimum follow-up period of 3 months. The inclusion criteria were as follows: (1) a diagnosis of CTS based on nerve conduction studies and electromyography; (2) positive results in Tinel's [16] and Phalen's tests [17]; (3) scheduled surgery to decompress the median nerve, and (4) at least 18 years of age. Patients with a positive history of autoimmune diseases, rheumatologic conditions, and previous traumas to the wrist were excluded. All subjects were Caucasian of Italian descent. The study protocol conformed to the tenets of the Declaration of Helsinki and was approved by the local ethics committee. Before the study, each participant was informed about the purpose of the study and signed informed consents were obtained.

### 2.2. Materials

Test materials were supplied by Agave Farmaceutici srl (Prato, Italy). The oral supplement tested in this study (Axin C) was in tablet form and contained alpha-lipoic acid (300 mg), curcumin phytosome (500 mg), and B-group vitamins (vitamin B1, 1.05 mg; vitamin B2, 1.2 mg; vitamin B5, 4.5 mg; vitamin B6, 1.5 mg). The concentrations of the actives were based on previous studies.

### 2.3. Procedures

The study participants (*n* = 180) were randomly divided into three study groups (*n* = 60 each). Patients in Group A served as controls and did not receive any supplementation either before or after surgery. Patients in Group B received one tablet twice a day both before and after surgery, for 3 months each (totaling 6 months of supplementation). Patients in Group C received one tablet twice a day before surgery only for a total of 3 months. A total of four assessment visits were performed as follows: *T*_0_, baseline visit three months before surgery; *T*_1_, visit immediately before surgery; *T*_2_, follow-up visit at 40 days; and *T*_3_, follow-up visit at 3 months.

### 2.4. Surgery

**Figure 1:**
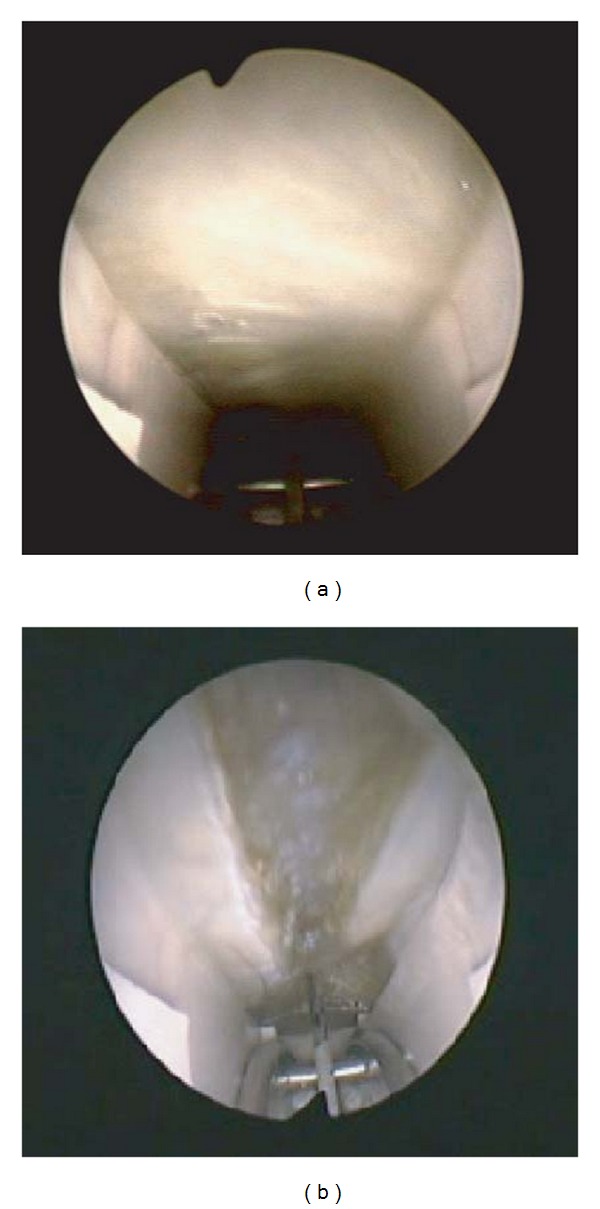
Progressive retrograde ligament section (panels a and b).

All patients underwent an endoscopic carpal tunnel release procedure under a same-day surgery regimen. Local anesthesia (without epinephrine) was performed with levobupivacaine 2% (5 mL) under median and ulnar nerve blocks at the wrist. A pneumatic tourniquet was positioned over the arm. A single-portal endoscopic carpal tunnel release system (Arthrex Inc., Naples, FL, USA) was used for this study. A single short (1 cm) transverse incision was performed at the level of the distal cutaneous crease on the volar side of the wrist. The incision was situated 1 cm proximal to the pisiform and 1 cm medially to the radial side of the wrist. After opening the brachial fascia proximal to the transverse carpal ligament and entering into the carpal tunnel space with a dissector, the endoscope was introduced and the white, transverse fibers of the ligament were visualized. Under clear vision, the ligament was cut by a retrograde release blade located at the extremity of the optic. A complete section of the ligament was confirmed after the detection of a “U” image in the monitor (Figure 1). Such an approach drastically reduced the risk of incomplete dissection of the ligament. The wound was closed with steri-strip and a compressive dressing was applied around the wrist and removed seven to ten days from surgery. All of the patients were encouraged to use their operated hand immediately on the first day from surgery, while avoiding wet and heavy burdens. Physiotherapy was undertaken in all patients to restore hand strength and ability.

### 2.5. Study Endpoints

The primary endpoint of the study was the magnitude of symptoms reduction (hyperalgesia, paresthesiae, nocturnal symptoms, and limitations to everyday life) in the three study groups. Each endpoint was evaluated at each visit on a 6-point Likert scale ranging from 0 (minimum) to 5 (maximum). Secondary endpoints were the numbers of positive and negative results in Tinel's and Phalen's tests, compliance, and patients' subjective satisfaction with the oral supplementation. 

### 2.6. Statistical Analysis

Categorical variables were assessed as counts and percent frequency and compared using the chi-squared test. The data were checked for normality using the Kolmogorov-Smirnov test for continuous variables. Normally distributed variables were compared across different time points (*T*_0_, *T*_1_, *T*_2_, and *T*_3_) using one-way analysis of variance (ANOVA) followed by post hoc Newman-Keuls tests. Skewed variables were analyzed using the nonparametric Kruskal-Wallis test followed by post hoc Tukey's test. Analysis of covariance (ANCOVA) was used to adjust for potential confounders. All statistical analyses were performed using the SPSS 17.0 package (SPSS Inc., Chicago, IL, USA). A two-tailed *P* value < 0.05 was considered statistically significant. Owing to the exploratory nature of the study, Bonferroni's correction was not applied.

## 3. Results

### 3.1. General Characteristics at Baseline

The baseline characteristics of the three study groups at *T*_0_ are shown in Table 1. The treatment groups were generally well matched. Specifically, the three groups were similar in terms of age, sex, hyperalgesia, paresthesia, and positive results in Tinel's and Phalen's tests at baseline. However, nocturnal symptoms and limitations to everyday life at *T*_0_ were found to be significantly higher in patients in Group B than in the other study groups. The study sample at baseline may be considered representative of a CTS population in need for surgical intervention.

### 3.2. Primary Endpoints

**Figure 2:**
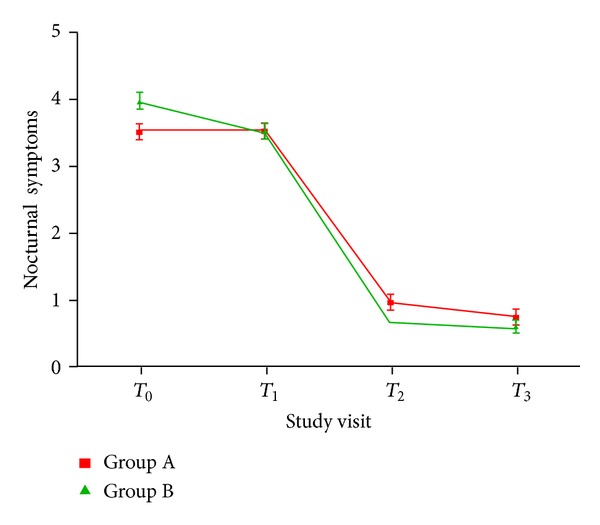
Changes in nocturnal symptoms observed in Groups A and B in the four study visits. The results indicated that patients in Group B had significantly lower nocturnal symptoms scores compared with Group A subjects at both *T*_2_ and *T*_3_ visits (both *P* values < 0.05). Interestingly, this effect was evident despite the higher baseline (*T*_0_) nocturnal symptoms scores in Group B patients.

The efficacy data for the primary study endpoints are shown in Table 2. The results indicated that patients in Group B had significantly lower nocturnal symptoms scores compared with Group A subjects at both *T*_2_ and *T*_3_ visits (both *P* values < 0.05). Interestingly, this effect was evident despite higher nocturnal symptoms scores in Group B patients at baseline (Figure 2, *P* < 0.05, ANCOVA after adjustment for potential confounders). However, no significant differences were found between Group A and Group C.

### 3.3. Secondary Endpoints

Table 3 shows the numbers of positive results in Tinel's and Phalen's tests. The results indicated that patients in Group B had a significantly lower number of positive Phalen's tests at *T*_3_ compared with Group A (*P* < 0.05). However, we found no significant differences between Group A and Group C. The compliance (assessed at the *T*_3_ visit) was 100% in 128 subjects (71.1%), 75% in 30 subjects (16.7%), 50% in 14 subjects (7.8%), and 25% in 8 subjects (4.4%). The overall satisfaction with oral supplementation was rated as excellent in 61 subjects (33.9%), good in 84 subjects (46.7%), average in 34 subjects (18.9%), and poor in 1 subject (0.5%). The supplement was well tolerated with no apparent adverse events.

## 4. Discussion

The results from the present study indicate that CTS patients who received oral supplementation with alpha-lipoic acid, curcumin phytosome, and B-group vitamins twice a day both before and after scheduled surgery, for 3 months each (totaling 6 months of supplementation), had a reduced burden of nocturnal symptoms (as assessed at 40 days post-surgery) and were less likely to have positive Phalen's test at 3 months after surgery. The treatment was associated with high satisfaction levels and good compliance.

In comparison with subjects suffering from radiculopathy (who generally show day-time pain with arm use), night pain is a common complaint of CTS patients [18, 19]. Importantly, the patient may be awakened with night-time pain, with a history of shaking the hand or flicking the wrist in an attempt to alleviate the discomfort [3]. There is also evidence suggesting that CTS patients who wake up at night because of pain are less likely to improve over time with treatment [3–5]. As night-time symptoms may be a sign of more severe cases of CTS, our data suggest that a combined supplementation approach with alpha-lipoic acid, curcumin phytosome, and B-group vitamins may have some value in reducing postsurgical night discomfort in CTS patients who underwent surgical decompression of the median nerve. It is worth noting that the benefits of oral supplementation were evident in patients who received the alpha-lipoic acid, curcumin phytosome, and B-group vitamins for six months despite having a higher baseline score of nocturnal pain before surgery. These data indicate that the beneficial effects on night-time pain observed in this study were not due to chance. Moreover, the results remained statistically significant even after adjustment for potential confounders in ANCOVA. Phalen's wrist flexion test is the most commonly reported provocative test for CTS and is generally the most widely accepted [17]. For this test, the patient allows the wrists to fall into full flexion letting the fingers dangle downward. If a tingling sensation in the distribution of the median nerve starts in less than one minute, it is considered a positive sign for the presence of CTS. The reduction rates of positive Phalen's test at 3 months after surgery were higher in subjects who received the oral supplementation for six months. These results demonstrate that the combination of alpha-lipoic acid, curcumin phytosome and B-group vitamins may be useful not only in reducing night discomfort but also in producing superior clinical results if started 3 months before decompression surgery and continued for 3 months thereafter. 

Previous studies have suggested that the neuroprotective effects of alpha-lipoic acid may limit and correct the clinical course of CTS. In a study of 112 subjects with moderately severe CTS, Di Geronimo et al. [9] reported a significant reduction in both symptoms scores and functional impairment after a 90-day treatment with a fixed association of alpha-lipoic acid and gamma-linolenic acid. In addition, supplementation resulted in a statistically significant improvement of electromyographic findings [9]. Several mechanisms may account for the potential clinical usefulness of alpha-lipoic acid in CTS, including its potent antioxidant activity [20] and its capacity to decrease neuronal sensitivity to pain by selectively inhibiting neuronal T-type calcium channels [21]. Moreover, animal studies have shown that alpha-lipoic acid may offer some protection against nerve ischemia and lipid peroxidation [22], help correct deficits in nerve blood flow [23], and improve distal sensory and motor nerve conduction [24].

Curcumin, a major active polyphenolic compound of turmeric (*Curcuma longa*), has been reported to have significant neuroprotective effects [25]. Curcumin not only acts as an effective antinociceptive agent [26] but also shows significant anti-inflammatory properties which include its inhibitory effects on the production of several inflammatory mediators (e.g., NF-*κ*B, IKK-*β*, COX-2, iNOS, TNF-*α*, and IL-6) [27]. The neuroprotective effects of curcumin have also been explained by possible protection effects against oxidative stress and the induction of antioxidative enzymes [25].

Although their effectiveness as a monotherapy is still controversial, B-group vitamins are often used as a conservative and adjunct therapy in the treatment of CTS. In a Cochrane review, Ang and coworkers [28] have critically analyzed the available evidence concerning the potential usefulness of B-group vitamins for treating peripheral neuropathies. The literature review concluded that there is moderate evidence that B-group vitamins at high doses may determine a significant short-term reduction in pain, numbness, and paresthesia in these clinical entities [28].

The results of our study indicate that the clinical benefits of supplementation with alpha-lipoic acid, curcumin phytosome, and B-group vitamins were evident only in patients who received the supplements twice a day both for 3 months before and a further 3 months after scheduled surgery (Group B). In contrast, we did not observe significant improvements in patients in Group C, who received oral supplementation twice a day for a total of 3 months before surgery only. These data indicate that maintaining supplementation after surgery clearly results in better outcomes as opposed to discontinuing treatment. We, thus, believe that the lack of adequate supplementation of CTS patients after surgery may lead to poor control of symptoms, lost productivity, reduced quality of life, and an increased incidence of complications. The management of CTS after surgery, thus, requires continued supplementation to improve the clinical outcomes. We also hypothesize that prolonged supplementation may improve symptoms by increasing blood supply to the nerve. In this regard, the blood supply needs of peripheral nerves are known to be related to their conducting function [29]. Notably, lipoic acid has been shown to increase the blood flow to the peripheral nerves [30].

There are several limitations to this study which need to be mentioned. First, this study was conducted in Caucasian individuals, so results cannot be simply extrapolated to populations with different ethnic backgrounds. Second, our study should be considered as an exploratory analysis and independent replication is needed to extend and confirm our results. CTS patients should be treated on an individual basis according to each patient's disease characteristics, based on clinical trial data and influenced by the personal experience of the surgeon. Third, although we showed that a combination treatment is effective in reducing nocturnal pain associated with CTS, our study did not compare the effect of the combination product with that of each compound alone (i.e., alpha-lipoic acid, curcumin phytosome, and B-group vitamins alone).

These caveats notwithstanding, the results of the present study suggest that CTS patients who receive oral supplementation with alpha-lipoic acid, curcumin phytosome, and B-group vitamins twice a day, for 3 months both before and after surgery, have a reduced burden of nocturnal symptoms at 40 days after surgery and are less likely to have a positive Phalen's test at 3 months after surgery. The treatment was associated with high satisfaction levels and good compliance, suggesting the potential clinical usefulness of this supplementation before and after surgery in CTS patients scheduled for the surgical decompression of the median nerve.

## Figures and Tables

**Table 1:** Baseline characteristics of the three study groups at *T*_0_ (three months before scheduled surgery).

Variable	Group A (*n* = 60), no treatment	Group B (*n* = 60), supplementation 3 months before and 3 months after surgery	Group C (*n* = 60), supplementation 3 months before surgery only	*P*
Age, years	60.1 ± 12.6	58.7 ± 14.1	55.0 ± 17.2	0.14
Male sex, (*n*)	12 (20%)	15 (25%)	17 (28.3%)	0.56
Hyperalgesia	2.57 ± 1.18	2.88 ± 1.41	2.58 ± 1.29	0.34
Paresthesia	3.12 ± 1.01	3.41 ± 1.30	3.12 ± 1.27	0.32
Nocturnal symptoms	3.52 ± 0.95	3.97 ± 0.95	3.55 ± 1.14	**<0.05**
Limitations to everyday life	2.17 ± 1.10	2.41 ± 1.22	1.72 ± 1.05	**<0.01**
Positive Tinel's test, (*n*)	60 (100%)	58 (96.6%)	57 (95%)	0.21
Positive Phalen's test, (*n*)	56 (93.3%)	51 (85%)	53 (88.3%)	0.34

Hyperalgesia, paresthesia, nocturnal symptoms, and limitations to everyday life were rated on a 6-point Likert scale ranging from 0 (minimum) to 5 (maximum). Data are expressed as counts and percentages or means and standard deviations, as appropriate. Statistically significant differences across the study groups are marked in bold.

**Table 2:** Primary study endpoints in the four study visits.

	Group A				Group B				Group C			
	*T*_0_	*T*_1_	*T*_2_	*T*_3_	*T*_0_	*T*_1_	*T*_2_	*T*_3_	*T*_0_	*T*_1_	*T*_2_	*T*_3_
Hyperalgesia	2.57 ± 1.18	2.75 ± 1.08	1.73 ± 0.88*	1.40 ± 0.84*	2.88 ± 1.41	2.59 ± 1.35	1.49 ± 0.90*	1.24 ± 0.79*	2.58 ± 1.29	2.48 ± 1.26	1.47 ± 0.91*	1.23 ± 0.79*
Paresthesias	3.12 ± 1.01	3.23 ± 0.98	1.13 ± 0.86*	0.83 ± 0.81*	3.41 ± 1.30	2.93 ± 1.29	0.90 ± 0.73*	0.78 ± 0.72*	3.12 ± 1.27	2.92 ± 1.27	0.93 ± 0.88*	0.80 ± 0.84*
Nocturnal symptoms	3.52 ± 0.95	3.52 ± 0.93	0.97 ± 0.90*	0.75 ± 0.88*	3.97 ± 0.95	3.53 ± 0.91	0.69 ± 0.70^∗†^	0.61 ± 0.77^∗‡^	3.55 ± 1.14	3.12 ± 1.13	0.77 ± 0.90*	0.70 ± 0.85*
Limitations to everyday life	2.17 ± 1.10	2.33 ± 1.08	1.43 ± 0.85*	0.83 ± 0.81*	2.41 ± 1.22	2.39 ± 1.05	1.31 ± 0.70*	1.07 ± 1.27*	1.72 ± 1.05	1.78 ± 0.92	1.20 ± 0.82*	0.92 ± 0.72*

Data are expressed as means and standard deviations. Statistically significant differences between Group A and Group B at the *T*_2_ and *T*_3_ visits are marked in bold. *P < 0.001 versus baseline. ^†^ *P* < 0.05 versus Group A at *T*_2_. ^‡^ *P* < 0.05 versus Group A at *T*_3_.

**Table 3:** Results of Tinel's and Phalen's tests (secondary study endpoints) in the four study visits.

	Group A				Group B				Group C			
	*T*_0_	*T*_1_	*T*_2_	*T*_3_	*T*_0_	*T*_1_	*T*_2_	*T*_3_	*T*_0_	*T*_1_	*T*_2_	*T*_3_
Positive Tinel's test, (*n*)	60 (100%)	60 (100%)	18 (30%)*	10 (16.6%)*	58 (96.6%)	57 (95%)	14 (23.3%)*	8 (13.3%)*	57 (95%)	57 (95%)	15 (25%)*	12 (20%)*
Positive Phalen's test, (*n*)	56 (93.3%)	57 (95%)	16 (26.6%)*	12 (20%)*	51 (85%)	51 (85%)	12 (20%)*	**4 (6.6%)** ^∗†^	53 (88.3%)	55 (91.7%)	13 (21.7%)*	8 (13.3%)*

Data are expressed as counts and percentages. Statistically significant differences between Group A and Group B at the *T*_3_ visit are marked in bold. *P < 0.001 versus baseline. ^†^ *P* < 0.05 versus Group A at *T*_3_.
